# Impact of prostate focused alignment on planned pelvic lymph node dose

**DOI:** 10.1002/acm2.13092

**Published:** 2021-07-07

**Authors:** Joshua Kilian‐Meneghin, Tianjun Ma, Lalith Kumaraswamy

**Affiliations:** ^1^ Roswell Park Comprehensive Cancer Center Buffalo NY USA

**Keywords:** alignment, radiotherapy, fiducial, lymph nodes, prostate, VMAT

## Abstract

**Purpose:**

Prostate patients with positive lymph node margins receive an initial course of 45 Gy to the planning target volume (PTV) comprised of prostate, seminal vesicles, and lymph nodes with a 1‐cm margin. The prostate is localized via implanted fiducial markers before each fraction is delivered using portal‐imaging. However, the pelvic lymph nodes are affixed to the bony anatomy and are not mobile in concert with the prostate. The aim of this study was to determine whether a significant difference in pelvic lymph node coverage exists between planned and delivered external beam therapy treatments for these patients.

**Methods:**

The recorded prostate motions were gathered for 19 patients; conjointly the pelvic lymph node motions were determined by manual registration of the bony anatomy in the kV‐images. The difference between the prostate and the bony anatomy coordinates was input into Eclipse as field shifts to represent the deviation in planned vs delivered pelvic lymph node coverage.

**Results:**

Structure volume at V(100) was recorded for each patient for two structures: summed pelvic lymph nodes (LN CTV) and pelvic lymph nodes +1 cm margin (LN PTV) to express their contribution to the PTV. For the LN PTV, the average difference between the planned coverage and calculated delivered coverage was 3.5%, with a paired *t*‐test value of *P* = 0.005. Based upon bony anatomy registration, 26% of patients received less than 95% dose coverage using V(100) criteria for LN PTV. Dose value differences between the two plans at minimum were 6.96 ± 6.23 Gy, at mean were 0.54 ± 0.40 Gy, and at maximum were 0.10 ± 0.29 Gy. For the LN CTV, the average difference between the planned coverage and calculated delivered coverage was 1%, with a paired *t*‐test value of *P* = 0.53.

**Conclusions:**

The results indicate a significant difference exists between the planned coverage and calculated delivered coverage for the LN PTV. There was no significant difference found for the LN CTV. We conclude that lymph node motion must be considered with the prostate motion when aligning patients before each fraction.

## 1 INTRODUCTION

### 1.A VMAT

The use of volumetric modulated arc therapy (VMAT) to treat prostate cancer allows for a higher dose gradient and decreased healthy tissue toxicity, when compared to three‐dimensional (3D) conformal radiation therapy. Volumetric modulated arc therapy also offers an improvement in speed compared to standard intensity modulated radiation therapy,^1^ with treatment times ranging from 3 to 5 min. Highly modulated VMAT plans require millimeter levels of accuracy while aligning the patient each fraction to deliver the prescribed dose to the target while minimizing the dose to the healthy surrounding organs. Prostate cancer is one such disease that would benefit from the aforementioned precision. The prostate is a difficult organ to localize, as it is moved on a daily basis by rectum and bladder filling. The National Cancer Institute’s SEER program (The Surveillance, Epidemiology, and End Results) estimates just over three in 25 men will be diagnosed with prostate cancer within their lifetime.^2^

### 1.B Treatment

Prostate cancer patients with positive lymph node margins receive an initial course of 45 Gy to the PTV comprised of prostate, seminal vesicles, and lymph nodes with a 1‐cm margin. The subsequent boosts are administered to the PTV that does not contain the lymph nodes, thus this study will compare initial 45 Gy courses exclusively. As discussed, prostate treatment has the added complication of movement. One method for localizing the prostate is to implant the patient with high‐Z fiducial markers and use portal imaging on the day of treatment to bring the prostate back into alignment with the treatment plan.^3^ Small movements of the prostate can be covered by the internal margin, and errors in setup by the setup margin; both of which are included in the planning target volume (PTV). This method ensures that the entire prostate gets the intended dose on every day of treatment. The current method assumes that all of the internal anatomy within the initial 45 Gy PTV moves rigidly around the prostate’s location. Since the pelvic lymph nodes are affixed to the bony anatomy, as noted by Hsu et al.,^4^ aligning with couch movements via the prostate coordinates could result in the lymph nodes receiving a different dose than what was conceived on the treatment planning system. Two possible solutions to the rigid motion assumption issue come from Thornqvist et al.,^5^ who demonstrated the feasibility of a deformable motion model to generate appropriate margins to insure coverage of all three PTV structures and by Hwang et al.,^6^ who demonstrated a method to adopt motion corrections with the multi‐leaf collimator and varying segment weighting in conjunction with fiducial marker prostate localization. Both studies do not quantify the loss in pelvic lymph node coverage due to the independent motion of prostate and the lymph nodes. Pang^7^ also notes that the intra fraction prostate motion increases over time, thus shorter duration treatments are preferred, and current margin standards can be inadequate.

The aim of this retrospective study was to determine whether a significant difference in pelvic lymph node coverage exists between planned and delivered external beam therapy treatments for the sample of patients who were aligned with implanted fiducial markers in the prostate.

## 2 MATERIALS AND METHODS

### 2.A Patient and course selection

Nineteen prostate cancer patients with positive lymph node margins, that received their full prescriptions, were selected for this retrospective study. The initial 45 Gy PTV for these patients comprised of the prostate, lymph nodes, and seminal vesicles. Later boosts have PTVs that no longer contain the lymph nodes. The study was conducted on the initial 45 Gy treatment to demonstrate the prostate alignment effect on lymph node dose.

### 2.B Structures of interest

**Fig 1:**
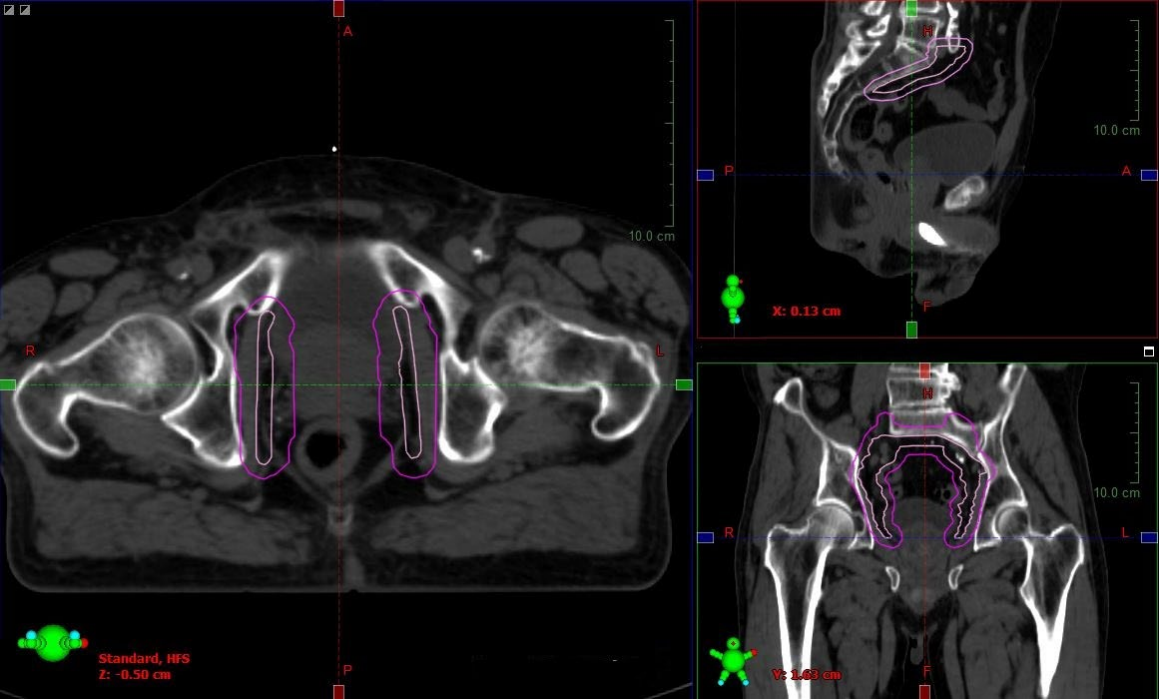
Contoured lymph node CTV (light pink) and LN PTV (dark pink).

The structures of interest to this study are those of critical importance to the treatment that do not move with the prostate. They are the lymph nodes and the lymph node contribution to the PTV. Lymph nodes are affixed to the bone and are therefore less susceptible to rectum and bladder induced motion. The lymph nodes were investigated to ensure that adequate dose was delivered to the lymph nodes (LN CTV) and the lymph node PTV (LN PTV). The initial 45 Gy PTV is created by adding 1.0 cm margin around the CTV comprised of prostate, seminal vesicles, and involved lymph nodes. To isolate the effects on the lymph nodes, a new structure was created by adding 1 cm margins to the lymph node contours alone, henceforth called LN PTV (Fig. 1). Lateral edges of the LN PTV were trimmed to match the 45 PTV dimensions if they were changed from the standard by the dosimetrist during the treatment plan process.

### 2.C Elekta Mosaiq

**Fig 2:**
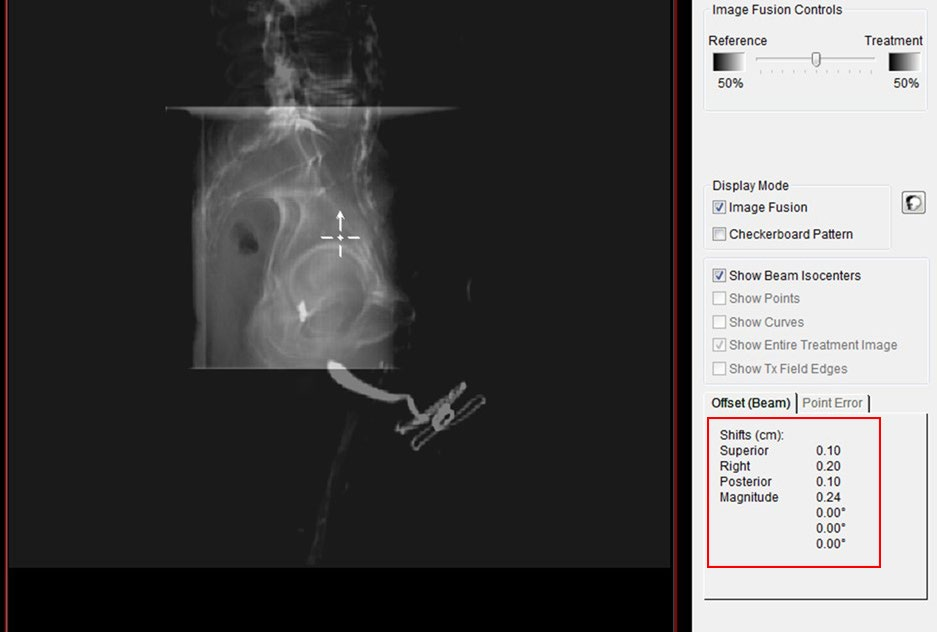
Mosaiq registration tool, with highlighted shift data.

**Fig 3:**
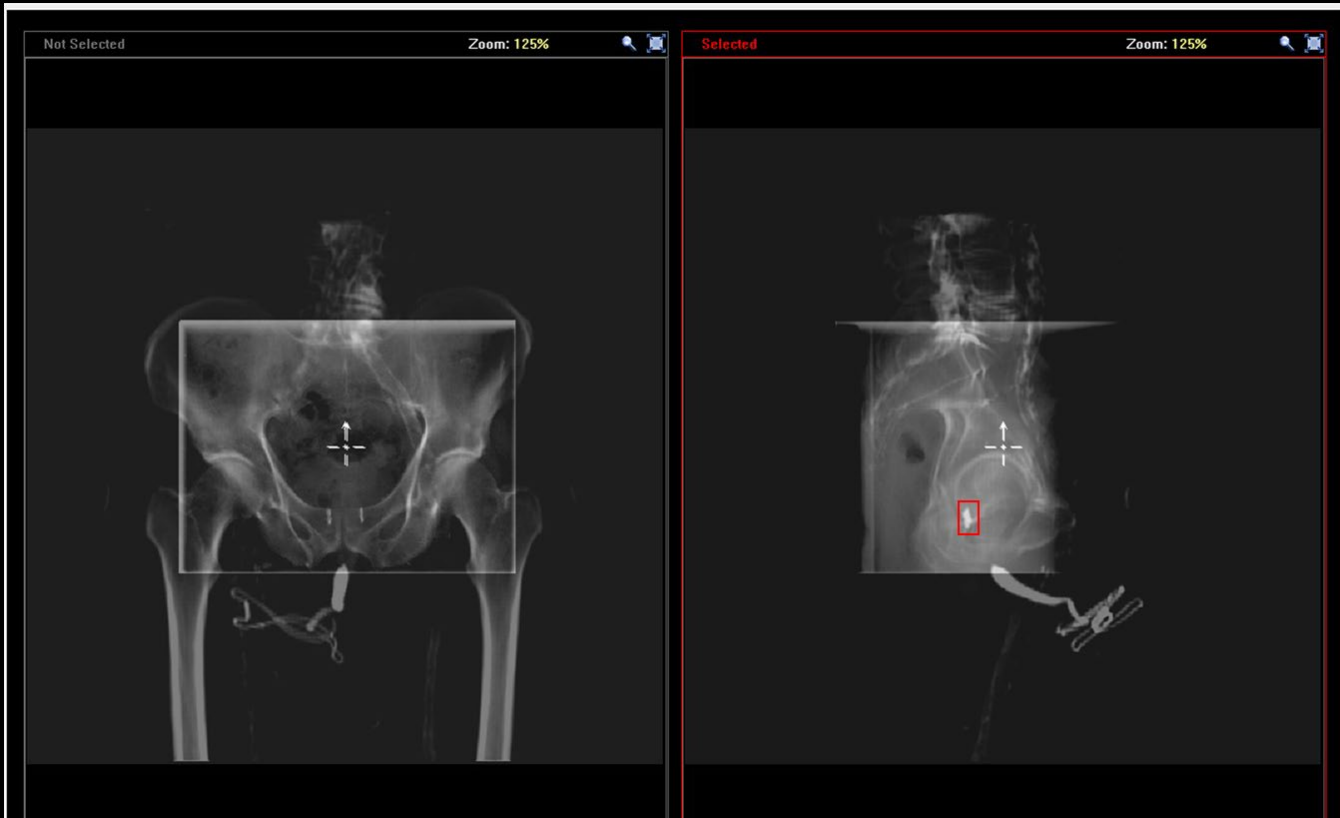
Mosaiq registration tool, with highlighted fiducial markers.

Elekta Mosaiq is used at our institution as the Record and Verify system. Mosaiq stores all the localization shift information from the daily kV‐kV alignment for all the patients as shown in Fig. 2. The prostate shift coordinates were extracted from the system for the first 25 fractions, for each of the 19 patients. These shift coordinates represent the localization of the initial PTV based on the implanted fiducial markers in the prostate. As mentioned earlier, the initial PTV also encompasses the involved lymph nodes, which is fixed to the bony anatomy rather than with the prostate. To determine the location of the involved lymph nodes during each fraction treated, the orthogonal kV images were manually aligned retrospectively using the pelvic brim as the matching structure, and those coordinates were extracted to determine the location of the lymph nodes in relation to the treatment isocenter as shown in Fig. 3.

### 2.D Coordinate systems

There are three coordinate systems that are used in this body of work to describe the location of structures due to patient motion. The first are the daily shift coordinates based on the implanted fiducial markers that describe the treatment couch movements required to bring the prostate back into the equivalent position of {0, 0, 0} in the Eclipse plan. If these coordinates were input into Eclipse as field shifts they would describe the location of the prostate before alignment was completed. The second set of coordinates are the manual bone alignment values. Using these values as couch shifts would bring the pelvic brim back into alignment with the Eclipse plan, on the day of treatment. Such a method would treat the bone‐affixed lymph nodes as the Eclipse plan intended, but at the expense of the prostate, which will be misaligned. Input of these values as field shifts into Eclipse will describe the location of the pelvic brim before realignment. The final set of coordinates are the lymph node misalignment values, which are calculated by taking the difference between the prostate shift coordinates and the bone shift coordinates. Using these values as couch shifts would describe the location of the pelvic brim on the day of treatment, after prostate alignment has been completed. Input of these values as field shifts in Eclipse would describe the misalignment of the bony anatomy, and the lymph nodes, when the treatment was delivered. The resultant dose calculations can then be compared to the original plan to describe the discrepancy in dose between the calculated original plan and the projected delivered dose, for structures affixed to the pelvic bones. All three sets are in units of centimeters and have twenty‐five subsets of {x, y, z} values (one for each fraction).

## 3 RESULTS

### 3.A Aggregate misalignment analysis

**Fig 4:**
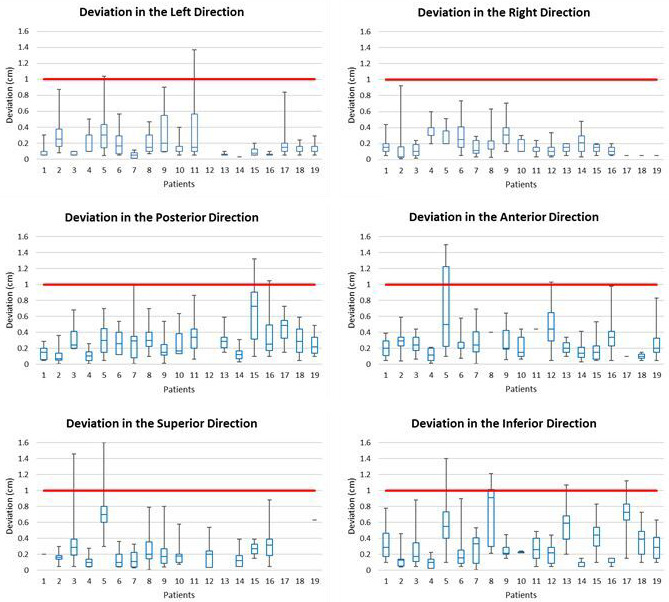
Minimum, maximum, and quartiles of misalignment for 19 patients, by patient, in six directions, with 1‐cm PTV margin for reference.

Figure 4 illustrates the lymph node misalignment values in each direction for each patient, which are created by taking the difference between the prostate shift coordinates and the bone shift coordinates as described in Section 2.D. Indicators with a Y‐value of 0 in Fig. 4 designate that the patient had no shift in the specified dimension. The inferior direction had four maximum deviations above the 1.0‐cm margin, which was the greatest number in any dimension, as well as the largest average deviation at 0.33 cm. The maximum distance between the prostate and lymph node alignment in the right direction for any patient did not deviate more than the 1.0‐cm margin, and the left direction had the lowest observed average deviation in any direction of 0.16 cm. In the posterior direction, patient #15 had the maximum misalignment of 1.32 cm and the average misalignment for all patients in this direction is 0.28 cm. A similar trend is seen in the anterior direction with the average deviation of 0.28 cm and a maximum misalignment of 1.5 cm (patient #5). The single largest maximum deviation observed in the study occurred for patient #5 in the superior direction, with the deviation of 1.6 cm. While is it important to note that all planes of movement, other than the right direction, had shifts larger than the 1 cm margin, of key significance is the 0.33 cm average shift in the inferior direction. This was the largest observed shift for the averaged nineteen patients and can be a contributing factor for the findings discussed in the subsequent investigations.

### 3.B LN PTV structure

**Fig 5:**
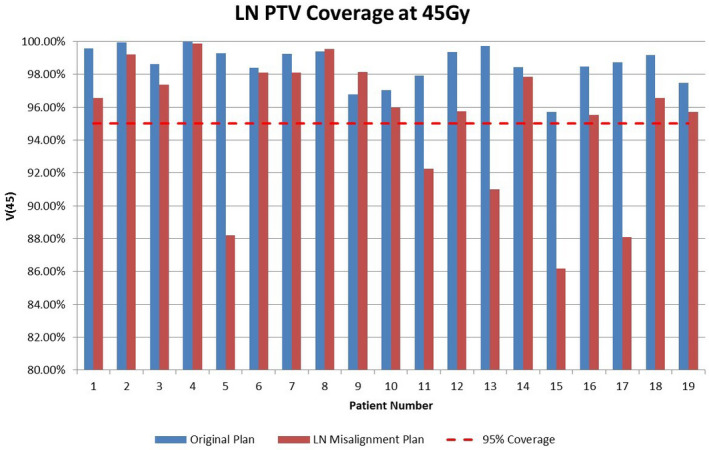
LN PTV volume receiving prescription dose (45 Gy) for the original plan and the LN misalignment plan, across 19 patients.

Figure 5 presents the results for the LN PTV structure at 45 Gy for the original and LN misalignment plan. This structure does not include the prostate’s contribution to the PTV. The LN misalignment plan is created by summing all the individual LN misalignment plans obtained for each fraction. Via this method, a patient’s 25 individual daily shifts are incorporated into the analysis. The individual LN misalignment plans are generated in Eclipse TPS by taking the difference between the prostate shift coordinates and the bone shift coordinates, as described in Section 2.D. The LN misalignment plan will be used to describe the calculated, predicted dose for the position of the lymph nodes after prostate alignment has occurred.

The absolute mean difference for V45 between the original and LN misalignment plans on average was 3.5%. The aforementioned paired t‐test value was *P* = 0.001, indicating strong significance and affirming that the difference between the two plans was not due to statistical fluctuations. This result signifies that the lymph node portion of the PTV in the average patient does not receive the planned dose, on average 3.5% less coverage at 45 Gy.

**Fig 6:**
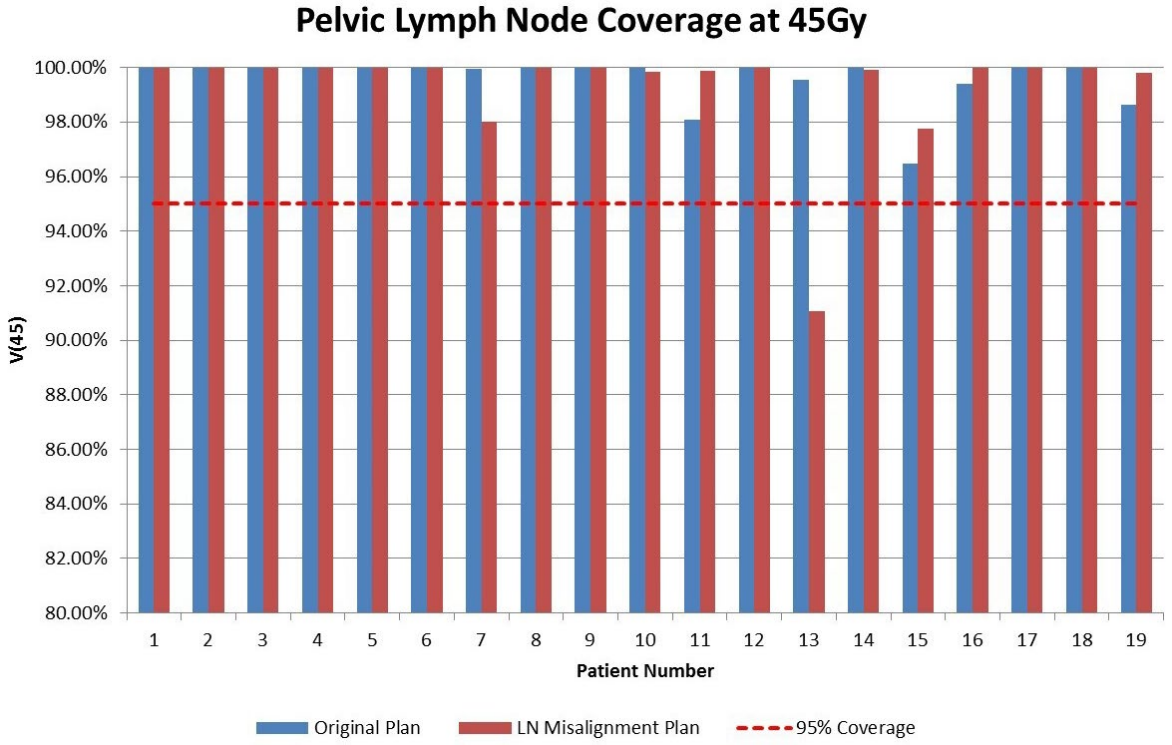
Lymph node volume receiving prescription dose (45 Gy) for the original plan and the LN misalignment plan, across 19 patients.

Without an explicit RTOG definition of the lymph nodes’ contribution to the PTV, an examination at the 95% coverage (V95) value can provide some meaningful analysis. All original plans received 95% coverage for the LN PTV. Five out of 19 patients received less than 95% coverage on the LN misalignment plan, which describes the location of the lymph nodes during treatment. Additionally, those five patients had an original LN PTV coverage over 95%, but the LN misalignment plan coverage was below that limit. This result describes cases where the planned lymph node dose met the criteria as a portion of the PTV in planning, but when treated was under‐dosed. The maximum difference between the two plans for one single patient for the LN PTV structure was 11% volume at 45 Gy. The average patient had a lymph node CTV of 305.8 cm^3^ and a LN PTV of 1056 cm^3^, or 345% of the CTV volume. This large difference in volume accentuates the loss of coverage displayed in Fig. 5 relative to that of Fig. 6 and highlights the impact of lymph node misalignment of the volumetric coverage metric used as the baseline to define an adequate dosage by organizations like the RTOG.

### 3.C Lymph node structure

Figure 6 displays the volume of lymph node (CTV) covered by the 45 Gy (V45) for the original and LN misalignment plans. There is no specific limit for the lymph node coverage, but clinically we expect V45 to be >95%. All the original plan cases meet the coverage expectation for the lymph node. All but one lymph node misalignment plans met the same coverage criteria. This patient (Patient 13) has a very long and narrow lymph node CTV compared to an average patient. Since most of the misalignment occurs in the inferior‐superior direction, even slight misalignments can cause large losses in dose coverage for the LN CTV. We noted that results for the LN PTV for patient 13 were very similar to that of the CTV (Fig. 5). This would indicate that for this patient’s anatomy, they would be vulnerable to motion induced misalignment; however, with the margin of the PTV, adequate coverage for the lymph nodes could be maintained.

When taken as a whole, the absolute difference in coverage volume at 45 Gy for the LN CTV between the two plans was 1.1%. A paired t‐test was chosen to test the significance of the difference between the two plans, due to the intertwined nature of the two coverage values. The lymph node t‐test resulted in a value of *P* = 0.53, which indicates no significant difference between the original plan and the lymph node misalignment plan for lymph node CTV coverage.

Another metric for comparing dose to plans in Eclipse is to look at the minimum, maximum, and mean dose to a structure. Table 1 lists the difference between those values for the two structures of interest: the pelvic lymph nodes (CTV) and the LN PTV. The absolute dose values for the CTV are interesting to note, but a significant difference between the plans was not proven to exist. The results for the lymph nodes with margin, however, should be noted. The minimum value for the two plans showed the greatest discrepancy, in this case 6.96 Gy. The average original plan for all patient had a LN PTV min dose of 94.4% of the prescription; the misalignment plan was only 79.9%. The maximum and mean values were similar for both plans, resulting in a difference of 0.54 and 0.1 Gy respectively. The standard deviations are markedly large, compared to the differences themselves, which is a result of the great variability in dose difference between the plans when comparing the 19 patients.

**Table 1:** Difference between the original and lymph node (LN) misalignment plans at the minimum, maximum, and mean values for the pelvic lymph nodes and the LN planning target volume (PTV), averaged across 19 patients.

	Dose value as a Percentage of 45 Gy		Original minus misalignment plan	
	Averaged original plan	Averaged misalignment plan	% diff between plans	SD (Gy)
LN minimum dose	96.23%	96.65%	−0.44%	1.09
LN PTV minimum dose	94.48%	79.86%	15.47%	6.23
LN maximum dose	108.73%	107.77%	0.88%	0.32
LN PTV maximum dose	109.70%	108.38%	1.20%	0.40
LN mean dose	104.09%	104.31%	−0.22%	0.27
LN PTV mean dose	103. 93%	103.66%	0.26%	0.29

### 3.D LN PTV V95 shift dependence

**Fig 7:**
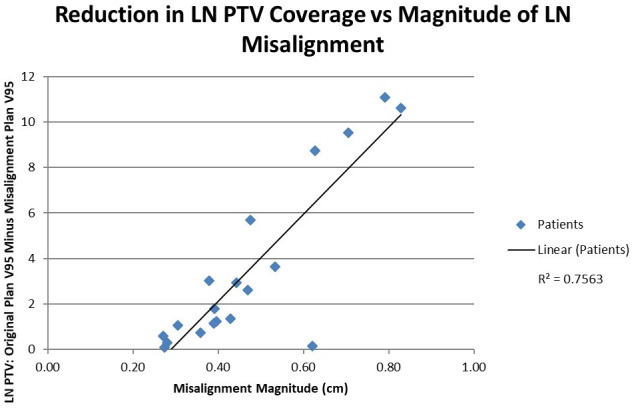
The difference between the volume receiving prescription dose (45 Gy) for the original plan and the LN misalignment plan, correlated with the magnitude of LN PTV misalignment, across 19 patients.

Ultimately, this study establishes the correlation between prostate shift magnitude and a reduction in calculated lymph node coverage, shown in Fig. 7. Referring to Fig. 5, the loss of coverage at V95 for the LN PTV structure can be quantified by taking the difference between the original plan’s coverage, and that of the misalignment plan. When plotted against the absolute average shift value, obtained in quadrature, for each patient, an interesting correlation emerges. As the magnitude of the average shift value increase, so does the loss of coverage. While the majority of the nineteen patients had both a moderate loss in coverage and average shift value, when taken as a whole a relatively strong (R^2^ = 0.76) linear trend is evident.

## 4 DISCUSSION

### 4.A LN PTV coverage and inferior‐superior shifts

The deviation trends show a significant lymph node misalignment in the inferior direction across the nineteen patients studied. The average misalignment in the inferior direction is 0.33 cm and had four maximum misalignments above the 1.0 cm margin level. When compared with the results displayed in Fig. 4, the inferior and superior shifts, noted in Fig. 7, patently drive the noted trend, and reinforce the conclusion that careful planning when selecting appropriate margins in the inferior‐superior dimension is required; inattention resulting in the above‐noted lack of lymph node coverage.

The RTOG protocol^8^ states that 98% of the total PTV will receive 100% of the prescription dose. For this initial course, all 19 patients were prescribed 45 Gy, in 1.8 Gy fractions. All patients tested in this study achieved this goal: greater than 98% planned coverage for the total PTV. However, not all had 100% coverage. The likely situation is that the prostate and seminal vesicles received at or near 100% coverage due to the realignment, but the lymph node portion of the PTV did not achieve the planned coverage. When the three structures are combined, the lymph node contribution to the PTV coverage is compensated for by the combined PTV: comprising of prostate and seminal vesicles. A weighted average of LN PTV and 54 PTV scaled with volume on average increased volume covered by 45 Gy by 1% on average in the original and misalignment plans (not displayed in Figs. 5 and 6). In all but two cases, which were most likely constrained by normal organ limits, this returns original plan coverage to at or above 98%. The PTV is intended to compensate for a loss of coverage around target structures due to motion, and it is apparent that it is functioning as intended, given the positive patient outcomes. However, when the planned dose is misaligned, in some cases by values larger than the margin, quality coverage can be sacrificed. Some patients had misalignments of the lymph nodes larger than the margins of the standard PTV. These effects caused 26% of the patients involved in the study to receive <95% dose coverage for the PTV, when separated into lymph node and prostate sections. Improved margin design using the results from this study could insure that every fraction meets the Eclipse plan fluence, instead of the majority of fractions.

### 4.B Sample size

This study was submitted as an AAPM abstract with a fifteen‐patient sample size. Additional patients could refine the shift analysis model for future work, thus four more patients were subsequently added to the study, bringing the total sample size up to 19 patients. All statistical conclusions were maintained: lymph node dose difference paired *t*‐test value went from *P* = 0.35 to *P* = 0.53, and LN PTV dose difference paired *t*‐test value went from *P* = 0.01 to *P* = 0.001. There is variability in the p‐values, but it is believed, based upon the new values, that adding more patients to the sample size will not change the conclusions defined in the study.

### 4.C Prospective solutions

This study discusses the planning consequences of operating under with the assumption that the lymph nodes move in concert with the prostate. Murray^9^ reviews the intrinsic threat of low coverage for prostate cancer patients: micro disease. The presences of lymph node micro disease impacts prudent treatment methods and negatively impacts outcomes.^10^ As shown in Figs. 5 and 6 all patients planned received adequate coverage for their CTVs, however, coverage was lacking in a statistically significant portion of their PTVs. We would like to emphasize that the misalignment plans were generated using the shift values to align the fiducial markers each day for the patients. The PTV is comprised of the internal margin and the setup margin. If the lymph node portion of that PTV is already compromised at the beginning of the treatment, then misalignments have the potential worsen within the fraction.^7^ There are several potential solutions: deformable registration with inter‐faction adaptive treatments, improvements to bladder filling protocol to total reduce motion, and an expansion of target margins in the most active regions of misalignment.^11^, ^12^, ^13^, ^14^ At our clinic we have adopted asymmetric margin expansions for the CTV to better account for the motion.^15^ In addition, CBCT scans are acquired in the daily set‐up process for the first 25 to facilitate improved lymph node alignment. For the subsequent fractions where the PTV no longer contains lymph nodes, fiducial markers are the primary source of alignment, which aligns with the findings of Clancy et al.^16^

## 5 CONCLUSION

Given the results observed by this study, the conclusion can be drawn that lymph node motion, or the lack thereof, must be taken into serious consideration when aligning patients. This study also establishes that the standard PTV margins effectively encompass the lymph node CTV, and therefore our institution sees good patient outcomes. However, there is room for improvement. The data acquired in this study can be used to design patient‐specific margins that more effectively reach target goals, while enabling reduced normal tissue toxicity.

## CONFLICTS OF INTEREST

Partial text submitted as “Impact of Prostate Focused Alignment On Pelvic Lymph Node Coverage” to the 59th Annual AAPM Meeting, SU‐I‐GPD‐T‐67, 2017.
